# Image Classification Using Biomimetic Pattern Recognition with Convolutional Neural Networks Features

**DOI:** 10.1155/2017/3792805

**Published:** 2017-02-16

**Authors:** Liangji Zhou, Qingwu Li, Guanying Huo, Yan Zhou

**Affiliations:** ^1^College of IOT Engineering, Hohai University, Changzhou 213022, China; ^2^Key Laboratory of Sensor Networks and Environmental Sensing, Hohai University, Changzhou 213022, China

## Abstract

As a typical deep-learning model, Convolutional Neural Networks (CNNs) can be exploited to automatically extract features from images using the hierarchical structure inspired by mammalian visual system. For image classification tasks, traditional CNN models employ the softmax function for classification. However, owing to the limited capacity of the softmax function, there are some shortcomings of traditional CNN models in image classification. To deal with this problem, a new method combining Biomimetic Pattern Recognition (BPR) with CNNs is proposed for image classification. BPR performs class recognition by a union of geometrical cover sets in a high-dimensional feature space and therefore can overcome some disadvantages of traditional pattern recognition. The proposed method is evaluated on three famous image classification benchmarks, that is, MNIST, AR, and CIFAR-10. The classification accuracies of the proposed method for the three datasets are 99.01%, 98.40%, and 87.11%, respectively, which are much higher in comparison with the other four methods in most cases.

## 1. Introduction

Image classification and recognition is a sophisticated task for machine, and it has been a hot issue in the field of Artificial Intelligence (AI) all the time. Feature extraction from an image is a significant step in automatic image classification. To effectively represent the image, many approaches have been proposed, and these approaches can be roughly categorized as hand-crafted features and machine learned features. The most representative hand-crafted features are scale-invariant feature transform (SIFT) [1] and Histogram of Oriented Gradient (HOG) [2]. These features are especially useful for the image classification on small-scale datasets. However, it is a too difficult problem to find proper features from images in the case of large-scale dataset. Moreover, the hand-crafted features are usually low-level features without enough mid-level and high-level information, which hinders the performance [3].

Over the last few years, Deep Neural Networks (DNNs) have achieved state-of-the-art performances in a wide range of areas [4–7]. Inspired by the mammalian visual system, Deep Convolutional Neural Networks (DCNNs) have become the most suitable architectures for many computer vision tasks [8]. CNNs, as generic feature extractors, have been continuously improving the image classification accuracy, avoiding the traditional hand-crafted feature extraction techniques in image classification problems. The features learned from CNNs are not designed by human engineers, but from data using a general-purpose learning procedure [9]. Because both hand-crafted features and machine learned features have their advantages, the reasonable combination of these two methods is becoming a hotspot recently [10–12].

A typical architecture of CNNs usually contains many layers to automatically extract useful image features and exploit the* softmax* function (also known as multinomial logistic regression) for classification [13, 14]. However, the softmax classifier often shows a low prediction performance [15]. Moreover, a higher precision gained by CNNs also means a deeper structure, more learning parameters, and larger amount of training data, leading to a cost of increased training complexity. In addition, because overly increasing depth can harm the accuracy, even if the width/filter sizes are unchanged, a deeper structure does not always guarantee a better result, which has been validated by many experiments [16].

To tackle the problem mentioned above, some viable research has already been proposed. If a well-performed classifier was added behind the CNN, the classification accuracy will be improved in some degree, and this is exactly the starting point of CNN-SVM. CNN-SVM is a combination of CNN and SVM [17], which take CNN as a trainable feature extractor and SVM as a classifier. Firstly, CNN is utilized to learn feature vectors from the image data. Then the learned vector representations are fed to a SVM classifier as features for image classification. It should be noted that, in the whole process, CNN and SVM are trained separately to get a better result [18]. The results provided by a combination of CNNs and SVM show higher accuracy rate compared with alone use of CNNs or SVM. The running time of the combination method is significantly lower than that of SVM. Inspired by its success, this kind of combination is also adopted by other studies [19, 20].

Biomimetic Pattern Recognition (BPR) [21] is a new model of pattern recognition, which is based on “matter cognition” instead of “matter classification.” This new model is much closer to the recognition function of human beings, who cognize matters class by class, than traditional statistical pattern recognition using “optimal separating” as its main principle. In the BPR, “cognizing” one class of matters is essential to analyzing and “cognizing” the shape of infinite points set made up of all samples of the same class. In a mathematical work [22] written by pre-Soviet academician, Aleksandrov pointed out “The concept of topological space is very general, and the science about topological space—topology—is the most general mathematical branch about continuity.” The mathematical tool of the BPR is just the method to analyze the manifold in point set topology. Therefore, the BPR is also called the Topological Pattern Recognition (TPR).

In this paper, a new method that combines CNNs with BPR is proposed to reduce the complexity of training networks and to improve the performance of classification. Because CNNs represent an inspiration of the cognitive neuroscience while BPR implies the cognitive psychology, it is reasonable to combine them together in the framework of cognitive science. In our framework, CNNs are used to automatically learn feature vectors from raw images, and then the learned feature vectors are projected into high-dimensional space to be covered by BPR classifier. Such a combination is expected to combine the advantages of CNNs on feature representation and BPR on classification. Meanwhile, an adaptive technique is adopted to tackle the problem of setting coverage radius in BPR classifier. Evaluations on MNIST, AR, and CIFAR-10 datasets show that the combined model excels the classic CNN, CNN combined with SVM, and Principal Component Analysis (PCA) combined with BPR models in classification accuracy.

The rest of the paper is organized as follows. In Section 2, we give some brief introduction of CNNs and BPR. In Section 3, the proposed CNN-BPR model is presented. In Section 4, three different benchmark datasets are used to validate the superiority of the proposed model. Finally, Section 5 gives the conclusion.

## 2. Related Works

### 2.1. Convolutional Neural Networks

The idea of CNNs was firstly proposed in [23] by Fukushima, developed in [24] by LeCun et al., and improved in [25, 26] by Simard, Cireşan, and others. GPU acceleration hardware has facilitated development of deep CNN (DCNN), which includes a deeper architecture with additional convolutional layers.

**Figure 1:**
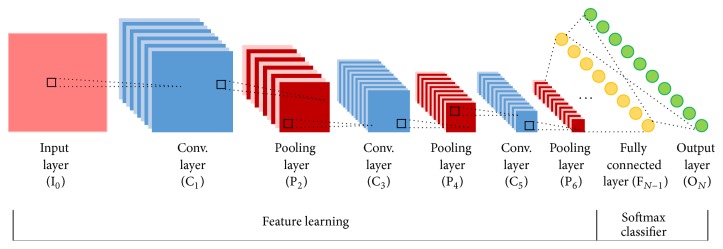
A typical convolutional network architecture.

Typical CNNs are composed of convolutional layer, pooling layer, and fully connected layer. A CNN consists of one or more pairs of convolution and pooling layers and finally ends with fully connected neural networks. Convolutional layers alternate with max-pooling layers mimicking the nature of complex and simple cells in mammalian visual cortex [27]. A typical convolutional network architecture is shown in Figure 1.

The 2D raw pixels of the image can be accepted as the input of CNNs directly. The image is then convolved with multiple learned kernels using shared weights. A convolutional layer is parametrized by the number of maps, the size of the maps, and kernel sizes. Each layer has* M* maps of equal size (*M*_*x*_, *M*_*y*_). A kernel of size (*K*_*x*_, *K*_*y*_) is shifted over the valid region of the input image. Each map in layer* l* is connected to all maps in layer *l* − 1. Neurons of a given map share their weights but have different input fields.

Next, the pooling layer reduces the size of the image while trying to maintain the contained information. The purpose of the pooling layers is to achieve spatial invariance by reducing the resolution of the feature maps. The output of the pooling layer is given by the maximum, mean, or stochastic activation, corresponding to max-pooling, mean-pooling, or stochastic-pooling, over nonoverlapping rectangular regions of size (*K*_*x*_, *K*_*y*_). Pooling creates position invariance over larger local regions and downsamples the input image by a factor of *K*_*x*_ and *K*_*y*_ along each direction. It turned out to be that max-pooling leads to faster convergence rate by selecting superior invariant features, which improves generalization performance [28].

Convolutional layer and pooling layer compose the feature extraction part. Afterwards, the extracted features are weighted and combined in one or more fully connected layers. This represents the classification part of the convolutional network. These layers are similar to the layers in a standard Multilayer Perceptron (MLP), where the outputs of all neurons in layer *l* − 1 are connected to every neuron in layer* l*.

Finally, there exists one output neuron for each object category in the output layer. The output layer has one neuron per class in the classification task. A softmax activation function is used; thus each neuron's output represents the posterior class probability.

For a *c* classification problem, it is standard for a CNN to use softmax or 1-of-*K* encoding at the top. Let* p*_*i*_ specify a discrete probability distribution, where *i* = 1,…, *c*, and ∑_*i*_^*c*^*p*_*i*_ = 1,** h** is the activation of the penultimate layer nodes,** W** is the weight vector between the penultimate layer and the softmax layer, the total input into a softmax layer, given by** a**, is

(1) $$\begin{array}{c} a_{i}=\sum_{k}h_{k}W_{ki}, \end{array}$$

then we have

(2) $$\begin{array}{c} p_{i}=\frac{\exp\left(a_{i}\right)}{\sum_{j}^{c}\exp\left(a_{j}\right)}, \end{array}$$

and the predicted class $\hat{i}$ would be

(3) $$\begin{array}{c} \hat{i}=\underset{i}{\arg\;\max\left(p_{i}\right)}=\underset{i}{\arg\;\max\left(a_{i}\right)}. \end{array}$$

CNNs are usually trained with a variant of the gradient-based backpropagation method [29]. All training patterns along with the expected outputs are fed into the network. Afterwards the network error (the difference between the actual and expected output) is backpropagated through the network and used to compute the gradient of the network error with respect to the weights. This gradient is then used to update the weight values according to a specific rule (e.g., stochastic, momentum, etc.) [30].

### 2.2. Biomimetic Pattern Recognition

The “biomimetic” emphasizes that the viewpoint of the function and mathematical model of pattern recognition is the concept of “cognition,” which is much closer to the function of human beings. An important and essential focus of attention in BPR is the principle of homology-continuity (PHC) [31].

**Theorem 1 .**

In the feature space *R*^*n*^, suppose that set *A* is a point set including all samples in class *A*. If *X*, *Y* ∈ *A* is given, there must be set *B*

(4) B=X1,X2,…,Xn ∣ X1=X,  Xn=Y,  n⊂N,  m⊂N,  ρXm,Xm+1<ε,  ∀ε>0,  1≤m≤n−1,B⊂A.

**Figure 2:**
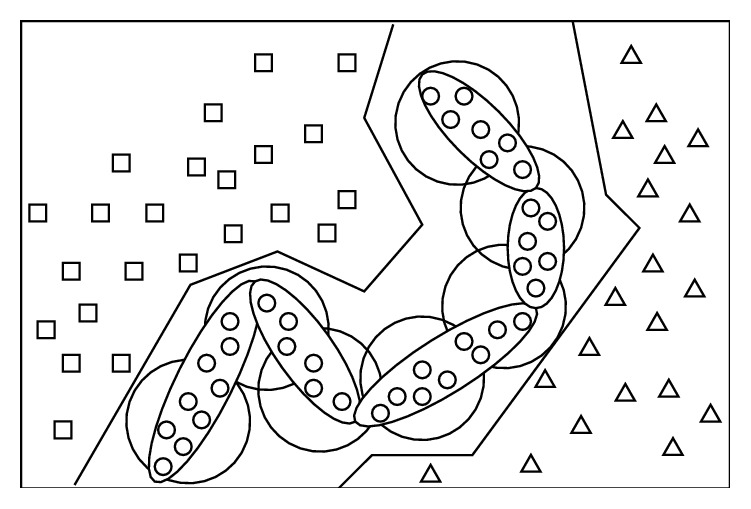
The schematic diagram of the difference of BP, RBF, and BPR.

“All useful information is included in the training set”—it is the basic of the traditional pattern recognition, but the theorem of PHC is beyond this hypothesis. The traditional pattern recognition is completely based on the separation of different samples in feature space because of the consideration that there is no a priori knowledge among the same sample points. However, “Universal Relation” is the objective law in nature, and it is followed by BPR, which makes full use of the law to improve the cognitive abilities of things.  Figure 2 shows the differences of the BPR and the traditional pattern recognition.

In Figure 2, the circles represent the samples to be recognized; the squares and triangles represent samples to be distinguished from circles. These small signs represent an idealized distribution of the sample points in feature space, and the polygonal line denotes the classification boundaries of traditional backpropagation (BP) networks, big circle denotes radial-basis function (RBF) networks (which is the same as the template matching), and the sausage-like shape represents “cognition” manner of BPR. The specific differences between BPR and the traditional pattern recognition are described in Table 1.

## 3. The Proposed Model

**Figure 3:**
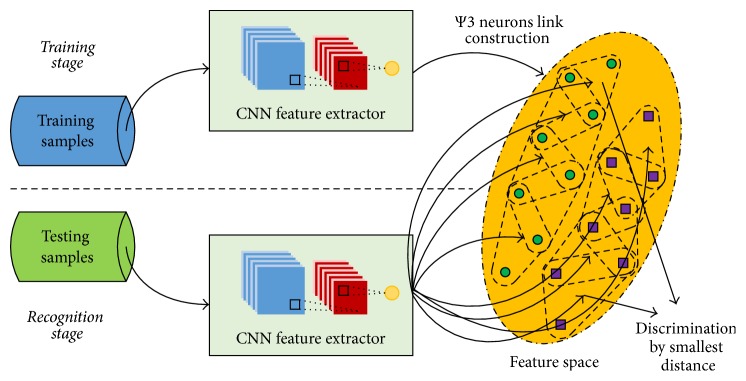
Flowchart of the proposed CNN-BPR recognition method.

In this section, we present a CNN-BPR combined model for image classification. The system framework is shown in Figure 3. Firstly, an automatic feature extraction is proposed by using CNN. Secondly, BPR is adopted as the classifier exploiting the features extracted from the previous module, CNN. To obtain the optimal coverage, an adaptive method is used to compute an appropriate coverage threshold for each class.

### 3.1. CNN-Based Feature Extraction

A CNN architecture with alternating convolutional and max-pooling layers is used here. Each node in the output layer corresponds to one character class. After CNN training, only the parameters of the fully connected layer are left to extract the final feature vector which will be fed to the BPR classifier. The CNN-based feature extractor that is irrelevant to the number of the character classes can be very compact. To extract the CNN-based feature, network training is used first. The training of CNN is composed of two main procedures, namely, forward propagation and backpropagation [32, 33].

#### 3.1.1. Forward Propagation of CNN

Assuming that layer *l* is a convolution layer and layer *l* − 1 is a subsampling layer or an input layer, the feature map* j* of layer* l* is calculated as follows:

(5) $$\begin{array}{c} x_{j}^{l}=f\left(\;\sum_{i\in M_{j}}x_{j}^{l-1}*w_{ij}^{l}+b_{j}^{l}\;\right), \end{array}$$

where *M*_*j*_ represents a selection of input maps, “*∗*” indicates the convolution computation; the essence of which is to convolve the convolution kernel **w** on all the associated feature maps of the layer *l* − 1; then sum them, together with the bias as the input of the activation function and finally get the output of convolution layer *l*.

A pooling layer produces downsampled versions of the input maps. If there are *N* input maps, then there will be exactly *N* output maps, although the output maps will be smaller. More formally,

(6) $$\begin{array}{c} x_{j}^{l}=f\left(\;\beta_{j}^{l}pool\left(\;x_{j}^{l-1}\;\right)+b_{j}^{l}\;\right), \end{array}$$

where **x**^*l*^ denotes the *l*-th subsampling layer, and pool(·) is pooling function which can be max-pooling, mean-pooling, or stochastic-pooling. Each output map is given its own multiplicative bias *β* and an additive bias *b*.

The procedure of forward propagation is composed of convolution and pooling alternately, and for full connection layer, all previous output maps are convoluted through each convolution kernel in this layer.

#### 3.1.2. Backpropagation of CNN

The training procedure of the CNN is the same as BP model. In the following, we define a squared-error function as the loss function *E*. For a multiclass problem with *N* training examples, *E* can be given as

(7) $$\begin{array}{c} E^{N}=\frac{1}{2}\overset{N}{\underset{n=1}{\sum }}{\left(\;t^{n}-y^{n}\;\right)}^{2}=\frac{1}{2}{\left\|\;t^{n}-y^{n}\;\right\|}_{2}^{2}, \end{array}$$

where *n* represents the *n*-th training example, *t*^*n*^ is its label, and *y*^*n*^ is the output.

However, different from the single structure of BP, there are differences in the training procedure for each layer of CNN. Here, we briefly present how to train parameters and compute the gradients for different types of layers. 

(*1) Convolution Layers.* The backpropagation “error” through the network can be considered as “sensitivity.” Assuming that each convolution layer *l* is followed by a subsampling layer *l* + 1, the residuals in the BP algorithm are equal to the weighted sums of the weights and residuals of all the nodes connected to the *l* + 1 layer and then multiplied by the derivative value of this point. The next layer of the convolution layer *l* is the subsampling layer *l* + 1, using one-to-one nonoverlapping sampling, so the residual calculation is simpler. The residual of the feature map *j* of the layer *l* is calculated as follows:

(8) $$\begin{array}{c} \delta_{j}^{l}=\beta_{j}^{l+1}\left(\;f^{\prime }\left(\;u_{j}^{l}\;\right)\circ up\left(\;\delta_{j}^{l+1}\;\right)\;\right), \end{array}$$

where “∘” denotes element-wise multiplication and up(·) denotes an upsampling operation the purpose of which is to extend the size of layer* l *+ 1 to the size of layer* l*, and it is also defined as the Kronecker product:

(9) $$\begin{array}{c} up\left(\;x\;\right)\equiv x⊗1_{n\times n}. \end{array}$$

Now given sensitivities map, the bias gradient is computed by simply summing over all the entries in **δ**_*j*_^*l*^.

(10) $$\begin{array}{c} \frac{\partial E}{\partial b_{j}}=\sum_{u,v}{\left(\;\delta_{j}^{l}\;\right)}_{uv}, \end{array}$$

Finally, to compute the gradients of the kernel weights, we sum the gradients for a given weight over all the connections that mention this weight:

(11) $$\begin{array}{c} \frac{\partial E}{\partial w_{ij}^{l}}=\sum_{u,v}{\left(\;\delta_{j}^{l}\;\right)}_{uv}{\left(\;p_{i}^{l-1}\;\right)}_{uv}, \end{array}$$

where (**p**_*i*_^*l*−1^)_*uv*_ is the* patch* in **x**_*i*_^*l*−1^ that was multiplied element-wise by **w**_*ij*_^*l*−1^ during convolution in order to compute the element at (*u, v*) in the output convolution map **x**_*i*_^*l*^. 

 (*2) Subsampling Layers.* For subsampling layer, the parameters of *β* and* b *are needed to learn. It is also worth noting that the proposed subsampling layer is following connected by convolution layer. The sensitivity of subsampling layer* l* is defined as

(12) $$\begin{array}{c} \delta_{j}^{l}=\overset{M}{\underset{j=1}{\sum }}\delta_{j}^{l+1}*w_{ij}, \end{array}$$

where **δ**_*j*_^*l*+1^ is the sensitivity of convolution layer *l* + 1. The additive bias is again the sum over the elements of the sensitivity map and can be rewritten (10) as

(13) $$\begin{array}{c} \frac{\partial E}{\partial b_{j}}=\sum_{u,v}{\left(\;\delta_{j}^{l}\;\right)}_{uv}. \end{array}$$

The multiplicative bias *β* involves the original downsampled map computed at the current layer during the feedforward pass. To compute the parameter *β*, we define

(14) $$\begin{array}{c} d_{j}^{l}=down\left(\;x_{j}^{l-1}\;\right). \end{array}$$

Then the gradient for *β* is given by

(15) $$\begin{array}{c} \frac{\partial E}{\partial \beta_{j}}=\sum_{u,v}{\left(\;\delta_{j}^{l}\circ d_{j}^{l}\;\right)}_{uv}. \end{array}$$

### 3.2. Classification Based on BPR

The process of BPR for classification mainly consists of constructing complex geometry coverage in high-dimensional space and discrimination based on minimum distance. Some associated theories will be introduced in the following context briefly.

#### 3.2.1. Theory of Complex Geometry Coverage in High-Dimensional Space

The BPR uses topological high-dimensional manifold theory as a mathematical tool and realizable method, which is also the reason why it is called TPR. High-dimensional manifold theory is used by BPR to study the topological properties of the similar samples in feature space. The method—“Complex Geometry Coverage (CGC) in high-dimensional space”—is used to study the samples' distribution in feature space and to give reasonable cover, so the samples can be cognized [34].

To form the CGC in high-dimensional space, the Multiweighted Neuron is specifically used to cover the Simplex one by one. The definitions of Simplex and Multiweighted Neuron are as follows.

**Definition 2 .**

Suppose *P*_0_, *P*_1_,…, *P*_*k*_, (*k* ≤ *n*) are some irrelevant points in *n*-dimensional Euclidean space *E*^*n*^, which is to say the vectors ${\vec{p}}_{i}=P_{i}-P_{0}$, (*i* = 1,2,…, *k*) are linearly independent, and then point set *Ω*_*k*_ = {*X*∣*X* = ∑_*i*=0_^*k*^*a*_*i*_*P*_*i*_, ∑_*i*=0_^*k*^*a*_*i*_ = 1, *a*_*i*_ ≥ 0} is a *k*-dimensional Simplex with *P*_0_, *P*_1_,…, *P*_*k*_ as its vertexes.

Simply, respectively, line segment, plane triangle, and tetrahedron can be regarded as a 1-dimensional, 2-dimensional, and 3-dimensional Simplex in the Euclidean space.

**Definition 3 .**

Suppose *V* is a polyhedron in feature space *E*^*n*^,   *x* ∈ *E*^*n*^/*V* and the distance between* x* and* V* meets

(16) $$\begin{array}{c} d\left(\;x,V\;\right)=\left\{\;d_{min}\;|\;d_{min}=min\left(\;d\left(\;x,y\;\right)\;\right),\;\forall y\in V\;\right\}. \end{array}$$

If *U* meets

(17) $$\begin{array}{c} U=\left\{\;x\;|\;x\in d\left(\;x,V\;\right)<\text{Th},\;x\in \frac{E^{n}}{V}\;\right\},\;\text{Th}>0. \end{array}$$

then *U* is called a coverage of polyhedron.

**Figure 4:**
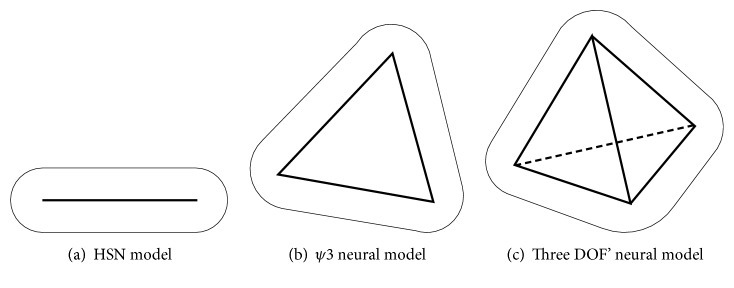
Some kinds of neural model of BPR.

When* V* is a line segment, a plane triangle, or a tetrahedron, the Multiweighted Neuron* U* is a Hyper Sausage Neuron (HSN), a *ψ*3 neuron, or a three degrees of freedom' (DOF) neuron, respectively.  Figure 4 shows a schematic representation of these types of neuron model.

#### 3.2.2. Recognition Algorithm Based on a Triangle Coverage

Suppose *S* = {*S*_1_, *S*_2_,…, *S*_*L*_} is a training set including* N* classes, and *S*_*k*_ = {*A*_1_, *A*_2_,…, *A*_*N*_} is the* k*th class which contains* N* sample points; here are the steps to construct CGC [35]:

**Step 1 .**

Calculate the distance *ρ* between any of two points in *S*_*k*_, and find two points* P*_11_ and* P*_12_ from the training set *S*_*k*_, and let *ρ*(*P*_11_, *P*_12_) = min_*A*_*i*_,*A*_*j*_∈*S*_*k*__{*ρ*(*A*_*i*_, *A*_*j*_)}, where *A*_*i*_ ≠ *A*_*j*_. Then find out a third point *P*_13_ ∈ *S*_*k*_ − {*P*_11_, *P*_12_}, where *P*_13_ is the nearest point away from* P*_11_ and* P*_12_ but not in line of them. Connect these three points {*P*_11_, *P*_12_, *P*_13_} to constitute the first plane triangle* T*_1_, which can be covered with a *ψ*3 neuron and the coverage space *θ*_1_ is

(18) $$\begin{array}{c} \theta_{1}=\left\{\;X\;|\;\rho_{XT_{1}}<Th,\;\;X\in R^{n}\;\right\}, \end{array}$$

where *ρ*_*XT*_1__ indicates the distance between* X* and* T*_1_, Th is the threshold, and the rest set *S*_*k*_ = *S*_*k*_ − {*P*_11_, *P*_12_, *P*_13_}.

**Step 2 .**

Judge whether or not points in* S* are in the coverage of *θ*_1_; if it is true, then exclude these points from* S* and let *S*_*k*_ = *S*_*k*_ − {*A*_*i*_∣*A*_*i*_ ∈ *θ*_1_}. Find out another point* P*_21_ from set *S*_*k*_ to make the minimum sum of distance from *P*_11_, *P*_12_, and *P*_13_. Rename the two points of {*P*_11_, *P*_12_, *P*_13_} as* P*_22_ and* P*_23_, which are the nearest two to *P*_21_, and then {*P*_21_, *P*_22_, *P*_23_} construct the second plane triangle* T*_2_. Likewise,* T*_2_ is covered with a *ψ*3 neuron generating the coverage space *θ*_2_, and the rest set *S*_*k*_ = *S*_*k*_ − {*P*_21_}.

**Step 3 .**

Find out other point *P*_*i*_ ∈ *S*_*k*_ as Step 2 does, marked as* P*_*i*1_, and the two nearest points are marked as* P*_*i*2_ and* P*_*i*3_. {*P*_*i*1_, *P*_*i*2_, *P*_*i*3_} construct the plane triangle *T*_*i*_ and further make the coverage *θ*_*i*_, and the rest set *S*_*k*_ = *S*_*k*_ − {*P*_*i*_}.

**Step 4 .**

Judge whether set *S*_*k*_ is empty; if it is true then end the construction; else repeat Step 3.

After the steps above, the eventual coverage of class* k* is the union of all coverage of neurons, which is

(19) $$\begin{array}{c} \Theta_{k}=\overset{m}{\underset{i=1}{⋃}}\theta_{i}. \end{array}$$

The basic recognition process is to judge which coverage the test sample would be covered with. Full coverage of training samples in different classes will inevitably result in overlapping space. A test sample might fall into none or overlapped coverage; then it belongs to the one closest to it. Therefore, the smallest distances need to be calculated between the test sample and each coverage. Let *ρ*_*i*_ be the distance between sample *X* and coverage space of class *i*; we have

(20) $$\begin{array}{c} \rho_{i}=\overset{M_{j}}{\underset{j=1}{min\;}}\rho_{ij},\;i=1,2,\ldots ,K, \end{array}$$

where *M*_*j*_ is the number of *ψ*3 neurons in class *i*, *K* is the total of classes, and *ρ*_*ij*_ is the distance between a sample to be recognized and the coverage of neuron *j* in class *i*. Then discrimination function is defined as

(21) $$\begin{array}{c} class=\arg\;\overset{K}{\underset{i=1}{\min\;}}\rho_{i},\;i=1,2,\ldots ,K. \end{array}$$

The pseudocode of CNN-BPR is given in Pseudocode 1.

## 4. Experiments and Discussions

To validate the proposed algorithm, three different datasets are used, namely, MNIST, AR, and CIFAR-10. Each dataset will be introduced briefly in the following paragraphs. For each group of experiments, the performance among CNN, CNN-SVM, PCA-BPR [36], HOG-SVM [37], and the proposed method is compared in the condition of different amount of training data. For CNN, we use the code downloaded from https://github.com/rasmusbergpalm/DeepLearnToolbox, and for the other three compared methods, they are all combined algorithms; we reimplement them according to the specific steps from their papers. The results demonstrate the validity of the proposed method.

### 4.1. Experiments on MNIST

**Figure 5:**
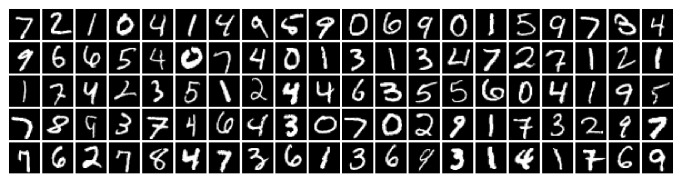
100 randomly selected images from MNIST.

The MNIST [38] dataset contains grayscale images of handwritten digits. Some images of the MNIST dataset are shown in Figure 5. It possesses ten different categories, namely, one for each digit from zero to nine. Each image has a fixed size of 28×28 pixels. The digits are centered inside the image and normalized in size. Totally, MNIST contains 70,000 images, including 60,000 training and 10,000 test images.

For MNIST, the input layer is followed by a convolutional layer C_1_ with 5 × 5 filters and 10 maps of size 24 × 24. The subsequent max-pooling layer P_2_ reduces the previous layer size to 12 × 12 by 2 × 2 filters. C_3_ also employs 5 × 5 filters but has 12 maps with dimensions of 8 × 8 pixels. P_4_ with 2 × 2 pooling windows yields 4 × 4 feature maps that are fully connected to 100 hidden neurons. These 100 hidden neurons are finally connected to the 10 output units. The structure of CNN can be briefly described as* 28 *×* 28Input-10C5-MP2-12C5-MP2-10Output*.

Because the CNN is employed as an automatic feature extractor and BPR as a classifier in our proposed method, after training for 20 epochs with a learning rate of 0.1, the fully connected layer with dimensions of 100 is projected into the feature space. Then, a series of *ψ*3 neurons are used to cover these feature points class by class.

**Figure 6:**
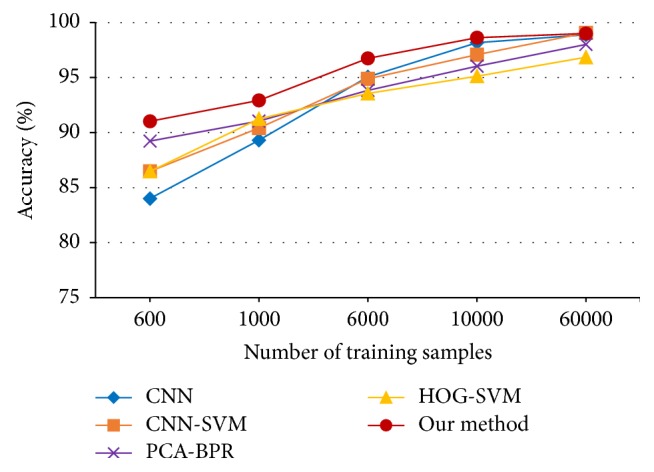
Classification accuracies versus number of training samples for MNIST.

CNN, CNN-SVM, PCA-BPR, and the proposed method are tested on the dataset. For CNN-SVM, we employ the 100 dimensional fully connected neurons above as the input of SVM, which is from LIBSVM with RBF kernel function. For PCA-BPR, same dimensional size of features are extracted from the top-100 principal components, and then *ψ*3 neurons are used to cover these feature points class by class. The experimental results are shown in Table 2. Moreover, in order to facilitate comparison with other methods, we set different numbers of training samples, which are 500, 1000, 6000, 10,000, and 60,000.  Figure 6 shows the comparison result.

### 4.2. Experiments on AR

**Figure 7:**
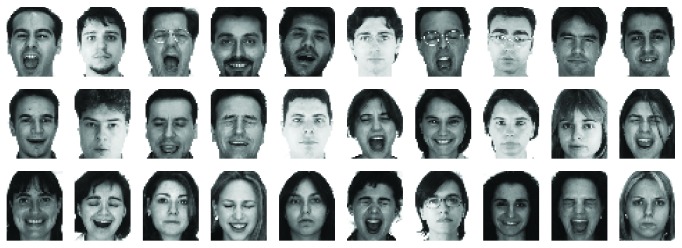
Sample images in AR dataset.

The AR database consists of over 3,200 frontal images of 70 men and 56 women, and there are 26 images of each individual [39]. The faces in AR contain variations such as illumination change, expressions, and facial disguises (i.e., sunglasses or scarf). We randomly selected 100 subjects (50 male and 50 female, 2,600 face images in total) in the experiments, and the images are cropped with dimension 165 × 120. For each individual, we select five images, totaled 500 face images for testing, and set different numbers of training samples by the rest images, which are 500, 800, 1,600, and 2,100. Some face images are shown in Figure 7.

**Figure 8:**
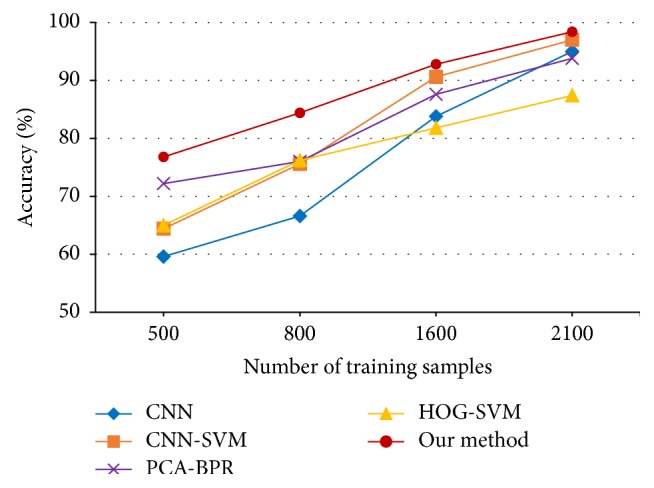
Classification accuracies versus number of training samples for AR.

The architecture of CNN is represented as* 165 *×* 120Input-20C5-MP4-50C5-MP2-80C3-MP2-120FC-100Output*, training 50 epochs with a learning rate of 0.01. Then the 120-dimensional feature points are covered by *ψ*3 neurons. The experimental results are shown in Table 3 and Figure 8.

### 4.3. Experiments on CIFAR-10

**Figure 9:**
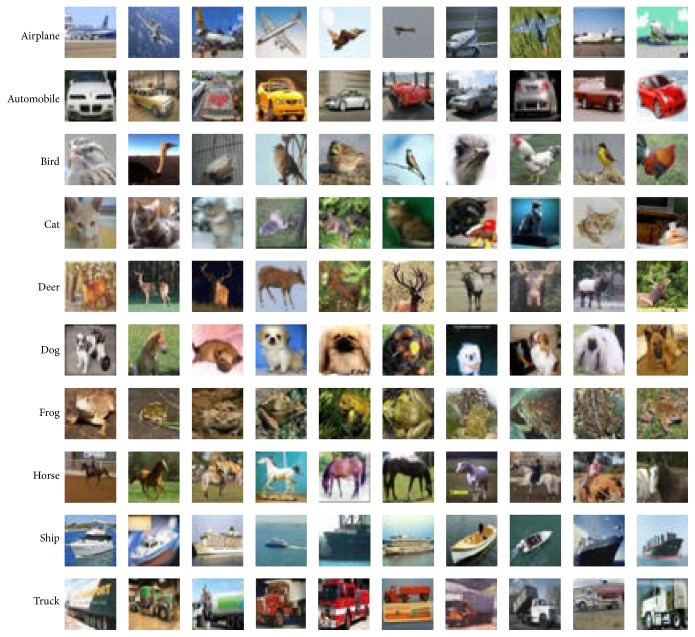
Sample images in CIFAR-10 dataset.

CIFAR-10 is a dataset of natural RGB images of 32 × 32 pixels [40]. It contains 10 classes with 50,000 training images and 10,000 test images. All of these images have different backgrounds with different light sources. Objects in the image are not restricted to the one at center, and these objects have different sizes that range in orders of magnitude. Some images of CIFAR-10 are shown in Figure 9.

**Figure 10:**
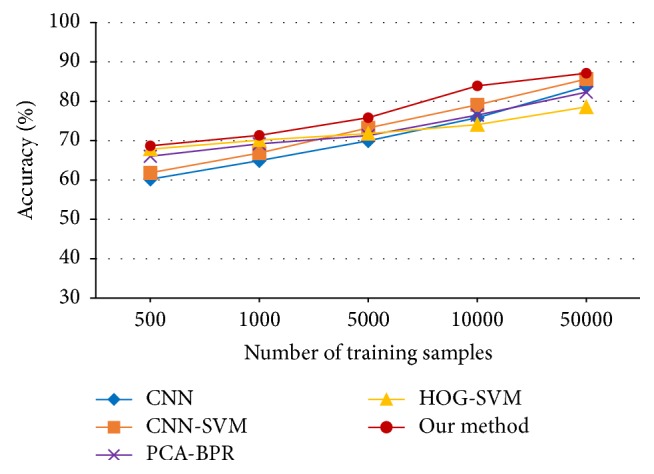
Classification accuracies versus number of training samples for CIFAR-10.

Because of RGB input images, there would be three channels in each filter for the first convolutional layer, which means the size of the filter would be 3 × 3 × 3 and three-dimensional convoluted with the input image, resulting in 12 maps of size 30 × 30 in layer *C*_1_. The following structure is* -MP2-16C3-MP2-120FC-10Output*, training 40 epochs with a learning rate of 0.1. Then the 120-dimensional feature points are covered by *ψ*3 neurons. The experimental results are shown in Table 4 and Figure 10.

From the above experiments, it can be seen that the CNN-BPR generally outperforms the other four methods. In the condition of the maximum training datasets, the CNN-SVM shows 0.17%, 2%, and 1.9% improvements compared to CNN, respectively, and CNN-BPR shows generally higher improvements of 0.12%, 3.4%, and 3.38% compared to CNN.

It can also be seen that HOG and BPR perform much better than the other methods in the case of small-sized homogeneous datasets, while with the increase of training samples, CNN-SVM surpasses the PCA-BPR, which means that CNN can better represent the feature than HOG and PCA do in the case of large-scale heterogeneous datasets.

## 5. Conclusion

In this paper, a CNN-BPR combined model for image classification is proposed. The proposed model treats CNN as a feature extractor, which can automatically learn the feature representation. BPR is adept in providing an accurate classifier. The results in terms of accuracy on the datasets of MNIST, AR, and CIFAR-10 show that the proposed method generally outperforms the other methods, which verify the effectiveness of the CNN-BPR combined image classification model.

Benefited from the unified framework of cognitive science, the combination of CNN and BPR represents a better performance than other methods. In the future, more choices of classification methods inspired by biology will be researched and compared in order to determine the best CNN-based framework for the image classification task.

## Figures and Tables

**Pseudocode 1:**
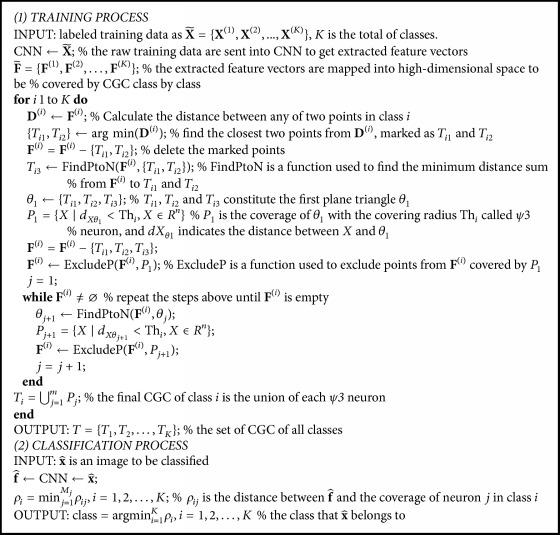
Pseudocode of the CNN-BPR algorithm.

**Table 1:** Comparison of traditional pattern recognition and BPR.

	Traditional pattern recognition	Biomimetic pattern recognition
Starting point	Optimal classification of different classes	Recognition of samples one by one class
Theoretical basis	All available information is included in the training set	Continuity of one sample class in feature space
Math tool	Statistics	Topology
Analyze methods	Theoretical derivation of algebra and equations (logical thinking)	Descriptive geometry of high-dimensional space (imagery thinking)
Recognition method	Division	Coverage of complex geometry in high-dimensional space
Realization approach	SVM and traditional neural networks	Multiweight high-order neural networks

**Table 2:** Classification accuracy of different methods on MNIST.

Training samples	Accuracy (%)				
	CNN [26]	CNN-SVM [19]	PCA-BPR [36]	HOG-SVM [37]	Our method
600	84.01	86.48	89.23	86.47	91.03
1000	89.31	90.41	91.04	91.24	92.93
6000	95.06	94.88	93.83	93.57	96.74
10000	98.17	97.09	96.03	95.13	98.62
60000	98.89	99.06	98.01	96.85	99.01

**Table 3:** Classification accuracy of different methods on AR.

Training samples	Accuracy (%)				
	CNN [26]	CNN-SVM [19]	PCA-BPR [36]	HOG-SVM [37]	Our method
500	59.6	64.4	72.2	65.0	76.8
800	66.6	75.6	76.0	76.2	84.4
1600	83.8	90.6	87.6	81.8	92.8
2100	95.0	97.0	93.8	87.4	98.4

**Table 4:** Classification accuracy of different methods on CIFAR-10.

Training samples	Accuracy (%)				
	CNN [26]	CNN-SVM [19]	PCA-BPR [36]	HOG-SVM [37]	Our method
500	60.19	61.83	66.03	67.82	68.68
1000	64.91	66.87	69.19	70.14	71.34
5000	69.94	73.20	71.26	71.76	75.85
10000	75.82	79.07	76.48	74.05	83.92
50000	83.73	85.63	82.29	78.56	87.11
